# The Gender Pain Gap: gender inequalities in pain across 19 European countries

**DOI:** 10.1177/1403494820987466

**Published:** 2021-02-10

**Authors:** Kweku Bimpong, Katie Thomson, Courtney L. Mcnamara, Mirza Balaj, Nasima Akhter, Clare Bambra, Adam Todd

**Affiliations:** 1 School of Pharmacy, Newcastle University, UK; 2 Population Health Sciences Institute, Newcastle University, UK; 3 Centre for Global Health Inequalities Research (CHAIN), Norwegian University of Science and Technology (NTNU), Norway; 4 Department of Anthropology, Durham University, UK

**Keywords:** Pain, inequality, socioeconomic factors, gender, Europe

## Abstract

**Aims:**

Chronic pain is increasingly considered to be an international public health issue, yet gender differences in chronic pain in Europe are under-examined. This work aimed to examine gender inequalities in pain across Europe.

**Methods:**

Data for 27,552 men and women aged 25–74 years in 19 European countries were taken from the social determinants of health module of the European Social Survey (2014). Inequalities in reporting pain were measured by means of adjusted rate differences (ARD) and relative adjusted rate risks (ARR).

**Results:**

At the pooled pan-European level, a greater proportion of women (62.3%) reported pain than men (55.5%) (ARD 5.5% (95% confidence intervals (CI) 4.1, 6.9), ARR 1.10 (95% CI 1.08, 1.13)). These inequalities were greatest for back/neck pain (ARD 5.8% (95% CI 4.4, 7.1), ARR 1.15 (95% CI 1.12, 1.19)), but were also significant for hand/arm pain (ARD 4.6% (95% CI 3.5, 5.7), ARR 1.24 (95% CI 1.17, 1.30)) and foot/leg pain (ARD 2.6% (95% CI 1.5, 3.8), ARR 1.12 (95% CI 1.07, 1.18)). There was considerable cross-national variation in gender pain inequalities across European countries.

**Conclusions:**

Significant gender pain inequalities exist across Europe whereby women experience more pain than men. The extent of the gender pain gap varies by country. The gender pain gap is a public health concern and should be considered in future prevention and management strategies.

## Background

‘Women get sicker, but men die quicker’ is a term used to describe gender inequalities in health, whereby women tend to have a higher life expectancy than men, although they live more of those years with morbidity [1]. In 2017, estimated life expectancies for people living in Europe were 83.5 years for women and 78.3 years for men, representing a gender gap of 5.2 years [2]. This ‘gender paradox’ is something that has been widely researched – with studies showing stark gender inequalities across various health outcomes and disease burden [3,4]. The reasons for this are complex, but it is thought that both sex (biological factors) and gender (social factors) play important, and interacting, roles. For example, increased incidence of osteoporosis in women can be largely explained by reduced levels of oestrogen associated with the menopause, while higher levels of depression amongst women are thought to have a genetic influence, as well as a social one (e.g. insecure life circumstances, feeling as though not in control, domestic and sexual violence) [5]. Social epidemiology has attributed some of women’s morbidity disadvantage – and men’s mortality disadvantage – to the restraints placed on women’s access to social and employment-related privileges and economic resources [6–8]. There is a lack of research examining gender inequalities in pain at a country level, especially across different European countries.

Chronic pain is something that is receiving increasing international interest from the public health community, not least due to the opioid epidemic. Chronic pain, defined as pain that persists for more than three months, is a global problem, and has significant impact on patients, their families, employers, health services and the wider economy. In the USA, for example, chronic pain is estimated to cost over US$500 billion annually: a cost exceeding the annual costs of heart disease, cancer and diabetes [9]. Similar challenges related to chronic pain have also been reported across Europe, where the most recent estimates suggest that up to 40% of the European population experience chronic back pain [10]. Recent estimates suggest that, in Denmark for example, one million working days are lost each year due to chronic pain [11].

The aetiology of chronic pain is complex and is thought to be influenced by a range of biological, social and behavioural factors. It is this complexity that makes chronic pain challenging to manage effectively, with many treatment strategies often relying on the use of opioid analgesics. At present, however, there are very few studies to support the long-term use of opioid analgesics in chronic pain management; opioids also cause adverse effects, including sleep disturbances, endocrine disorders, reduced immune function and increased pain through opioid-induced hyperalgesia. Indeed, the challenges of prescribing opioid analgesics in chronic pain have been well described by the US ‘opioid epidemic’, which has reported increasing levels of opioid misuse and overdose-related mortalities. The trend of increased prescribing of opioid analgesics in chronic pain management has also been reported across Europe [12,13]. Given this complexity, like other diseases, there is potential for gender differences in chronic pain prevalence, owing to different biological, social and behavioural factors. It is important to acknowledge and understand these potential differences so that appropriate treatment strategies can be developed, especially considering coping strategies and health-seeking behaviours may differ between men and women [14,15].

Given that the majority of the chronic pain epidemiology literature relates to the US situation, it is important for studies to explore chronic pain prevalence – and the associated inequalities – in other countries with different healthcare and welfare systems. An extensive European pain survey was published in 2006 by Breivik and colleagues [16], but the major focus of this work was on overall pain prevalence, rather than examining any gender inequalities in pain and how they might vary in different European countries. We have previously reported pain prevalence across Europe, as well as socioeconomic inequalities in pain [10]; however, this is the first paper to report on gender inequalities in pain across Europe.

## Aims

The aim of this study was to provide the first pan-European analysis of the gender inequalities in pain.

## Methods

### Data

This study was based on cross-sectional data concerning self-reported conditions from the ‘Social Inequalities in Health and their Determinants’ rotating module, which was included in the seventh round of the European Social Survey (ESS), 2014 [17,18]. The ESS is a cross-national survey conducted across Europe biennially that maps and illustrates interactions between institutional changes and social attitudes, beliefs and behaviour patterns. Data was obtained by presenting participants with a card listing several health problems, for example, muscular or joint pain in the back/neck, and were asked the following: ‘Which of the health problems on this card have you had or experienced in the last 12 months?’ Specifically, for the pain variables, survey participants could choose back/neck pain, arm/hand pain or foot/leg pain.

The health module used consisted of 40,185 respondents from 21 countries: Austria, Belgium, Czech Republic, Denmark, Estonia, Finland, France, Germany, Hungary, Ireland, Israel, Lithuania, Netherlands, Norway, Poland, Portugal, Slovenia, Spain, Sweden, Switzerland and the UK. The average response rate for all countries was 51.6%; Lithuania had the highest response rate with 68.9%, whereas Germany had the lowest with 31.4%. Data analysis was restricted to respondents aged 25–74 years to reflect ‘working age’. To ensure that the age exclusions did not impact on overall pain levels in the study population, we did a sensitivity analysis at the pan-European level comparing all ages (15+ years) to respondents aged 25–74 years (Supplemental Table 1).

Data concerning participants that did not have their gender or response to pain questions recorded were omitted from the analysis. Estonian responses were excluded from our analysis, as there was insufficient data relating to pain conditions reported. We also excluded Israel from our analysis, as it is not situated in Europe geographically, in line with previous work of Graham and colleagues [19]. After applying the above restrictions for in-eligible individuals and excluding cases for missing data (*n* = 383), our dataset included 27,552 participants (Supplemental Table 2).

### Variables

Data were analysed for three forms of pain: back/neck pain, arm/hand pain and foot/leg pain. We amalgamated results from these three pain variables and created a dichotomous variable to signify if participants had experienced at least one of the three forms of pain. RStudio (R v.3.5.1) was used to appropriately weight different populations and obtain pain prevalence estimates. Prevalence estimates were calculated for the pooled dataset and across all 19 European countries for which we had data.

### Analysis

Inequalities in reporting pain were measured by means of adjusted rate differences (ARDs) and relative adjusted rate risks (ARRs). Age-controlled ARDs and ARRs were calculated from predicted probabilities generated by means of binary logistic regression for pooled European data and country specific analysis. The ARD and ARR both express the relationship between two predicted probabilities based on observations and an estimated model. Stata 15.1 was used to conduct analyses concerning inequalities (obtaining ARD and ARR values); pain gender inequality was defined where both ARD and ARR were significantly different.

The ARD represents an absolute risk measure (absolute gender pain inequality/gap) and estimated whether the absolute difference is statistically different to zero. ARRs are calculated from predicted probabilities (the ratio of two probabilities); it compares the risk of a variable being present (taking place) when another variable (exposure) is present (relative gender pain inequality/gap). Here, we assessed the risk of pain among women in comparison to the risk of pain among men (reference group). An ARR value of > 1 indicated higher risk among women, whereas < 1 indicated lower risk among women. Data pertaining to ARDs and ARRs were input into Arc Map (v.10.5) in a comma-separated values (CSV) format. Shape files sourced from the Nomenclature of Territorial units for Statistics (NUTS) were attached to attribute data to produce choropleth maps using 10 equal interval categories.

### Weights

Age was controlled with reference to 10 age groups that consisted of 5-year intervals. These age intervals were weighted in accordance with the European Standard Population (ESP) [20]. To calculate pooled weights, the population size weight (pweight) which corrects for different population sizes between countries was combined with the post-stratification weight (pspweight) which uses information on age group, gender, education and region to reduce the sampling error and potential non-response bias of the survey. For country-specific estimates, only the pspweight was used.

## Results

### Pan-European level

Our findings show that a notable percentage of men and women across Europe experience pain. The prevalence of pain varied largely between different countries, as did the degree of gender inequality in pain. Overall, a greater proportion of women (62.3%) reported pain than men (55.5%), which was observed across all three-pain variables: the most common location was back/neck pain, experienced by 47.3% of women and 40.8% men. Then foot/leg pain, experienced by 26.6% of women and 24.3% of men, while 27.4% of women and 22.8% for men experienced hand/arm pain (Table I).

**Table I.** Prevalence of pain by gender in 19 European Countries.

Pain variable			Back/neck (%)	Hand/arm (%)	Foot/leg (%)	Any pain (% overall)
**Europe (pooled)**		Men	40.8	22.8	24.3	55.5
		Women	47.3	27.4	26.6	62.3
**North**	Denmark	Men	49.5	25	24.1	65.6
		Women	52.1	29.9	33.3	66.4
	Finland	Men	48.6	24.9	30.4	66.1
		Women	58.6	25.6	32.9	75.7
	Norway	Men	40.0	19.5	22.5	58.4
		Women	47.5	31.2	31.5	65.4
	Sweden	Men	43.0	24.1	24.6	60.1
		Women	51.6	29.1	25.4	66.3
**West**	Austria	Men	32.2	14.1	17.3	44.0
		Women	36.5	16.2	13.8	45.3
	Belgium	Men	50.0	25.3	25.1	65.0
		Women	55.7	31.0	29.0	69.7
	Switzerland	Men	37.8	17.3	24.4	54.4
		Women	43.6	20.8	20.7	58.0
	Germany	Men	48.6	21.5	24.8	62.1
		Women	59.6	26.3	27.7	70.5
	France	Men	46.6	28.7	28.1	62.2
		Women	54.9	35.0	26.2	73.1
	Ireland	Men	20.0	9.2	12.6	31.0
		Women	24.3	12.4	13.8	34.6
	Netherlands	Men	37.3	20.0	20.1	53.8
		Women	44.6	21.6	22.4	61.5
	UK	Men	38.8	22.7	26.9	56.4
		Women	39.1	24.0	27.5	57.9
**Centre/east**	Poland	Men	30.7	23.0	22.0	47.3
		Women	38.2	25.6	22.2	53.9
	Slovenia	Men	38.1	22.0	17.2	49.6
		Women	46.7	21.2	22.5	49.6
	Lithuania	Men	25.9	9.9	11.9	34.0
		Women	27.4	11.2	13.9	37.6
	Czech Republic	Men	21.3	9.5	10.6	31.1
		Women	29.1	14.7	16.7	40.0
	Hungary	Men	16.5	11.6	14.9	25.2
		Women	17.2	14.5	17.3	28.7
**South**	Portugal	Men	44.0	22.0	27.3	59.3
		Women	50.7	40.2	36.6	68.4
	Spain	Men	33.9	20.2	21.3	50.6
		Women	48.9	33.1	30.9	64.4

Prevalence’s were weighted using European Social Survey post-stratification weights and adjusted to the standard European population in accordance with the European Standard population (ESP) of 2013. Source: European Social Survey, 2014 [18].

**Figure 1.**
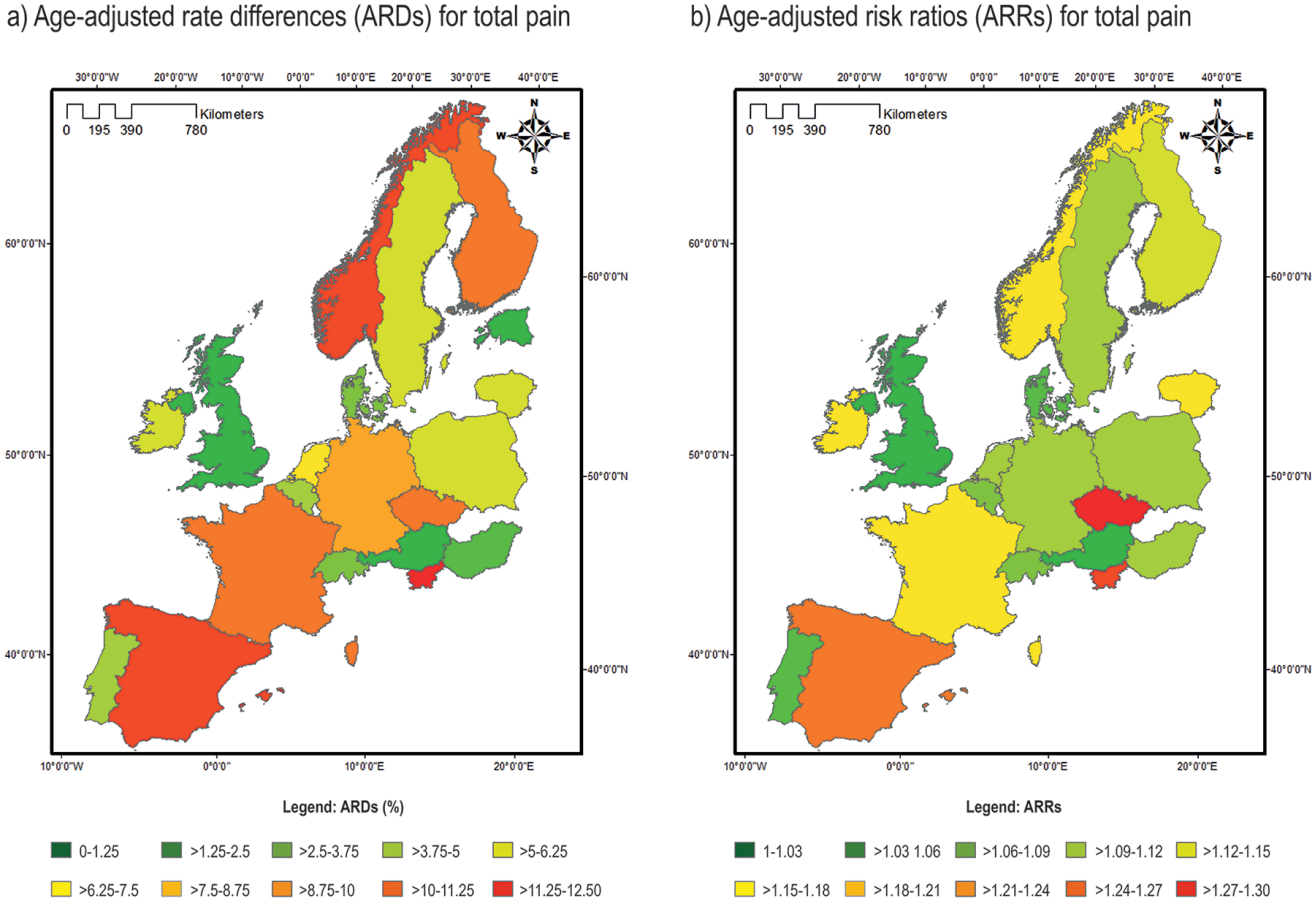
A map illustrating (a) age-adjusted rate differences (ARDs) and (b) age-adjusted risk ratios (ARR) in pain between men and women across Europe, with men as the reference category.

At a pan-Europe level, when examining all pain variables, gender pain inequalities were seen in both absolute and relative terms, with women more likely to report pain (ARD 5.5% (95% confidence intervals (CI) 4.1%, 6.9%) and ARR 1.10 (95% CI 1.08, 1.13)) (Table II and visually in Figure 1). These inequalities were greatest for back/neck pain (ARD 5.8% (95% CI 4.4, 7.1) and (ARR 1.15 (95% CI 1.12, 1.19)), but were also significant for hand/arm pain (ARD 4.6% (95% CI 3.5, 5.7) and ARR 1.24 (95% CI 1.17, 1.30)) and foot/leg pain (ARD 2.6% (95% CI 1.5, 3.8) and ARR 1.12 (95% CI 1.07, 1.18)).

**Table II.** Age-adjusted rate differences (ARDs) and age-adjusted rate ratios (ARRs) for gender inequalities in back/neck pain, hand/arm pain and foot/leg pain in 19 European Countries. Pain in men was the reference group. ARD estimated whether absolute difference is statistically different to 0, while ARR assessed if the rate ratio is significantly different to 1 (where 1 is equal risk). Gender pain inequality was defined where the ARD and ARR were both statistically significant.

Pain variables		Back/neck pain		Hand/arm		Foot/leg		Total pain	
		ARD (% (95% CI))	ARR (95% CI)	ARD (% (95% CI))	ARR (95% CI)	ARD (% (95% CI))	ARR (95% CI)	ARD (% (95% CI))	ARR (95% CI)
Europe	19 countries	5.8 (4.4, 7.1)	1.15 (1.12, 1.19)	4.6 (3.5, 5.7)	1.24 (1.17, 1.30)	2.6 (1.5, 3.8)	1.12 (1.07, 1.18)	5.5 (4.1, 6.9)	1.10 (1.08, 1.13)
North	Denmark	3.4 (–3.2, 10.0)	1.07 (0.94, 1.22)	4.6 (–1.3, 10.5)	1.18 (0.95, 1.47)	8.6 (2.7, 14.5)	1.35 (1.10, 1.70)	3.0 (–3.1, 9.2)	1.05 (0.95, 1.15)
	Finland	11.3 (5.7, 16.9)	1.23 (1.11, 1.37)	3.9 (–1.5, 9.2)	1.16 (0.95, 1.42)	3.0 (–2.3, 8.4)	1.10 (0.93, 1.30)	9.9 (5.0, 14.9)	1.15 (1.07, 1.23)
	Norway	11.0 (4.7, 17.2)	1.28 (1.11, 1.47)	11.2 (5.3, 17.1)	1.47 (1.20, 1.79)	11.3 (5.5, 17.1)	1.50 (1.22, 1.84)	10.7 (4.8, 16.7)	1.18 (1.08, 1.30)
	Sweden	8.2 (2.4, 14.0)	1.19 (1.05, 1.34)	7.5 (2.2, 12.7)	1.30 (1.08, 1.57)	3.1 (2.1, 8.2)	1.12 (0.92, 1.37)	6.2 (0.8, 11.7)	1.10 (1.01, 1.20)
West	Austria	3.5 (–1.6, 8.5)	1.11 (0.95, 1.29)	2.3 (–1.4, 6.1)	1.17 (0.90, 1.51)	–1.7 (–5.4, 2.1)	0.90 (0.70, 1.15)	0.9 (–4.3, 6.2)	1.02 (0.90, 1.15)
	Belgium	5.7 (–0.1, 11.4)	1.11 (1.00, 1.24)	6.1 (0.8, 11.4)	1.24 (1.03, 1.50)	4.1 (–1.0, 9.0)	1.16 (0.96, 1.41)	5.0 (–0.3, 10.2)	1.07 (1.00, 1.16)
	Switzerland	5.9 (0.3, 11.6)	1.16 (1.01, 1.33)	3.9 (–0.6, 8.4)	1.22 (0.97, 1.54)	–3.9 (–9.7, 1.0)	0.84 (0.68, 1.04)	3.7 (–2.0, 9.6)	1.07 (0.97, 1.18)
	Germany	9.1 (4.4, 13.7)	1.17 (1.08, 1.28)	4.8 (0.6, 8.9)	1.21 (1.03, 1.44)	3.2 (–1.0, 7.4)	1.13 (0.97, 1.32)	7.6 (3.3, 11.9)	1.12 (1.05, 1.19)
	France	7.4 (0.7, 14.0)	1.2 (1.01, 1.31)	5.8 (–0.5, 12.1)	1.20 (0.98,1.46)	–3.3 (–9.4, 2.8)	0.89 (0.71, 1.12)	9.9 (3.8, 16.0)	1.16 (1.06, 1.27)
	Ireland	3.5 (–2.2, 9.1)	1.13 (0.92, 1.38)	1.8 (–2.1, 5.7)	1.17 (0.83, 1.65)	3.0 (–1.0, 7.0)	1.26 (0.92, 1.72)	6.1 (0.3, 11.9)	1.18 (1.00, 1.38)
	Netherlands	8.3 (2.4, 14.2)	1.22 (1.06, 1.42)	1.6 (–3.1, 6.2)	1.08 (0.85, 1.37)	0.5 (–4.4, 5.3)	1.02 (0.81, 1.30)	6.6 (0.6, 12.6)	1.12 (1.01, 1.24)
	UK	–0.8 (–6.0, 4.5)	0.98 (0.86, 1.12)	–1.1 (–5.5, 3.3)	0.95 (0.79, 1.15)	0.0 (–4.7, 4.8)	1.00 (0.84, 1.19)	0.2 (–5.1, 5.5)	1.00 (0.91, 1.10)
Centre/ east	Poland	6.7 (1.2, 12.1)	1.22 (1.03, 1.43)	0.6 (–4.1, 5.2)	1.03 (0.83, 1.27)	–0.2 (–4.7, 4.2)	0.99 (0.80, 1.24)	5.7 (–0.1, 11.5)	1.12 (1.00, 1.27)
	Slovenia	10.9 (3.8, 18.0)	1.29 (1.09, 1.54)	1.4 (–4.6, 7.4)	1.07 (0.81, 1.40)	4.0 (–1.5, 9.5)	1.22 (0.93, 1.60)	12.4 (5.3, 19.4)	1.25 (1.09, 1.42)
	Lithuania	3.5 (–2.2, 9.1)	1.13 (0.92, 1.38)	1.8 (–2.1, 5.7)	1.17 (0.83, 1.65)	3.0 (–1.0, 7.0)	1.26 (0.92, 1.72)	6.1 (0.3, 11.9)	1.18 (1.00, 1.38)
	Czech Republic	7.4 (2.7, 12.1)	1.35 (1.11, 1.64)	5.2 (–1.5, 8.9)	1.55 (1.13, 2.13)	5.9 (2.1, 9.6)	1.58 (1.17, 2.12)	8.8 (3.7, 13.8)	1.29 (1.11, 1.49)
	Hungary	0.5 (–4.2, 5.2)	1.03 (0.77, 1.39)	2.5 (–1.8, 6.7)	1.22 (0.86, 1.71)	2.0 (–2.7, 6.7)	1.14 (0.84, 1.55)	2.5 (–3.0, 7.9)	1.10 (0.89, 1.34)
South	Spain	12.7 (7.4, 18.1)	1.36 (1.19, 1.55)	12.5 (7.8, 17.2)	1.61 (1.34, 1.94)	8.3 (3.6, 13.0)	1.38 (1.15, 1.66)	11.2 (5.8, 16.5)	1.22 (1.10, 1.34)
	Portugal	–0.4 (–9.0, 8.3)	0.99 (0.83, 1.18)	20.6 (12.9, 28.3)	1.85 (1.44, 2.38)	10.5 (2.8, 18.2)	1.37 (1.08, 1.74)	4.1 (–4.0, 12.2)	1.06 (0.94, 1.20)

### Country level

At an individual country level for the pooled pain variables, a gender pain gap was observed in most countries, with nine countries showing significant gender inequalities for total pain (for which we define as a significant difference in ARD and ARR). The gender pain gap was greatest in Slovenia (ARD 12.4% (95% CI 5.3, 19.4) and ARR 1.25 (95% CI 1.09, 1.42)) according to absolute measures. According to relative measures, however, the gender pain was greatest in the Czech Republic (ARD 8.8% (95% CI 3.7, 13.8) and ARR 1.29 (95% CI 1.11, 1.49)). Overall, for the pooled analysis, there were no significant gender pain inequalities in relative or absolute measures reported in Denmark, Austria, Belgium, Switzerland, UK, Poland, Hungary and Portugal.

In terms of inequalities for each pain variable, there were significant gender pain inequalities observed in 11 countries for back/neck pain; Spain had the highest gender pain inequalities in both relative and absolute terms (ARD 12.7% (95% CI 7.4, 18.1)) and ARR 1.36 (95% CI 1.19, 1.55)), followed by Finland in absolute terms (ARD 11.3% (95% CI 5.7, 16.9)) and ARR 1.23 (95% CI 1.11, 1.37)) and the Czech Republic in relative terms (ARD 7.4% (95% 2.7, 12.1) and ARR 1.35 (95% CI 1.11, 1.64)). No significant gender pain inequalities in relative or absolute measures were reported for Denmark, Austria, Belgium, Ireland, UK, Lithuania, Hungary and Portugal.

For hand/arm pain, significant gender pain inequalities were observed in 6 countries, with Portugal showing the highest pain gap in both relative and absolute terms (ARD 20.6% (95% CI 12.9, 28.3) and ARR 1.85 (95% CI 1.44, 2.38)), followed by Spain (ARD 12.5% (95% CI 7.8, 17.2)) and ARR 1.61 (95% CI 1.34, 1.94)). No significant gender pain inequalities in relative or absolute measures were reported for Denmark, Finland, Austria, Switzerland, France, Ireland, Netherlands, UK, Poland, Slovenia, Lithuania and Hungary.

For foot/leg pain, significant gender pain inequalities were observed in 5 countries; Norway had the highest inequalities using absolute measures (ARD 11.3% (95% CI 5.5, 17.1) and ARR 1.50 (95% CI 1.22, 1.84)), while Czech Republic had the highest inequalities using relative measures (ARD 5.9% (95% CI 2.1, 9.6) and ARR 1.58 (95% CI 1.17, 2.12)). No significant pain inequalities in relative or absolute measures were reported for Finland, Austria, Belgium, Switzerland, Germany, France, Ireland, Netherlands, UK, Poland, Slovenia, Lithuania and Hungary.

## Discussion

This paper used data from the seventh wave of the ESS from 2014 to establish the extent of gender pain inequality in 19 European countries. We have identified several key findings that may be important for researchers, practitioners and policy makers. Firstly, at the pan-European level, there are significant gender pain inequalities, with women more likely to experience pain than men, across all the three pain variables. Secondly, gender pain inequalities were more common for back/neck pain, with 11 out of 19 European countries showing significant inequalities, compared to hand/arm pain and foot/leg pain where only six and five countries respectively showed significant gender pain inequalities. Thirdly, the magnitude of the gender pain inequalities differed between European countries, with some countries exhibiting no gender pain divides.

Our work adds to the international literature on gender and health that shows women experience more morbidity than men. Research has found that although life expectancy is lower for men, women’s advantage does not translate into healthier years [21]. Women steadily report worse health status than men and suffer from a higher burden of non-fatal and debilitating conditions, such as arthritis, depression, or mobility problems [22]. Our research supports this by demonstrating significant gender inequalities in pain. Further, our work substantially extends the emerging international literature on the epidemiology of pain and inequalities in pain [10,23]. Previous research has similarly found that the prevalence of pain is higher in women; our work shows this is also the case in Europe [24–26]. Previous work using the ESS has reported on socioeconomic inequalities in pain prevalence across Europe and showed that the inequalities were most pronounced for hand/arm pain and least pronounced for back/neck pain [10]. Research has also found that women have higher rates of severely and moderately limiting pain as well as greater pain intensity [23].

The reasons for these gender inequalities are complex and not completely understood, although there appears to be a biological basis that helps to account for these gender differences: for example, sex hormones, such as oestrogen, can influence pain sensitivity [27]. Similarly, among different animal models, it has been shown that female rodents are more sensitive to chemical, heat and electrical signals than males [28–30]. Animal models have also shown that females have lower levels of stress-induced analgesia compared to males. Although these biological differences are well characterised, they do not fully explain our findings, given it is likely that pain sensitivity is also mediated by social and economic factors. Indeed, social epidemiological research has found that national and cross-national social policies implemented to support women and to promote gender equality (e.g. increasing gender equality in access to jobs, income, use of time, division of care work and political representation) are associated with improved health outcomes for women or decreased gender health inequalities [31–33]. This may also be the case for chronic pain, and is something that future research could examine.

Our work also has important public health policy and healthcare practice implications: pain and gender inequalities in pain are not a marginal issue and should be acknowledged in any pain treatment strategies going forward especially as increased pain is associated with higher opioid use [23]. Women are therefore potentially more at risk from the multiple side effects of opioid treatments (which include sleep disturbances, endocrine disorders, reduced immune function and increased pain through opioid-induced hyperalgesia). Whilst little can be done about the biological factors associated with higher pain prevalence (e.g. hormone levels), there is potential to change social circumstances in order to reduce the gender pain gap and to provide additional support/tailor treatments to women. Given the magnitude of the gender pain inequalities was highly variable between European countries, there may be opportunities to reduce these inequalities through health care or other measures. Future work could explore this further.

This study provides a unique insight into gender pain inequalities at a pan-European level using a comparative and robust dataset. However, our work should be considered in view of several limitations, as described below. For a detailed discussion regarding the strengths and limitations of the ESS data, see the work by Eikemo and colleagues [17]. Regarding our choice of model, we acknowledge that the use of logistic regression models has limitations for comparing outcome across groups due to the unobserved differences between groups. We intended to examine the inequality in pain prevalence between male and females, across European countries and Linear Probability model (LPM) are also suitable for this purpose [34]. To ensure our methodology was appropriate, we estimated the coefficients from the LPM across 19 European countries and Europe overall. We then compared these data with findings from binary logistic regression, which showed results of the logistic regression and LPM were in broad agreement (Supplemental Table 3).

A further limitation is that pain measures included in the survey were self-reported; as such, it was the sole responsibility of the participant to correctly identify if they experienced pain in the last 12 months, participant responses were not verified using medical records. Although we do accept that people who experience pain may not necessarily seek medical treatment, meaning that not all types of pain will be able to be verified from medical records. Further, we did not explore the intensity, severity, or type of pain in the survey so we did not consider this in our analysis. It is also possible that people might respond to the questions on pain differently (e.g. people with different cultural backgrounds). Caution is also recommended when translating pain estimates produced by our work into statements concerning the wider population, as our data was obtained using a survey as opposed to examining registry data. Whilst precaution was taken to avoid bias through the use of weightings, the survey selection technique may have resulted in a non-representative sample, as those with severe health conditions may have been unable to partake in the survey. As well as that, the ESS only samples from the non-institutionalised population thus potentially leading to bias, given the institutionalised population are more likely to be affected by poor health. We also note that response rates varied from country to country: while the ESS sets out 70% response rate targets, there were several countries that did not reach this target, although it is worth noting that this is not a direct indicator of a poor-quality dataset. Although the ESS collects data from 19 European countries covering all regions, there were several European countries not covered in the survey. Thus, our findings cannot be generalised to all European countries. Finally, the primary focus of our work aimed to establish if there were gender inequalities in pain prevalence across European countries; we did not seek to explain or account for these inequalities in our analysis. Future work should be more explanatory in nature and seek to examine how different healthcare and welfare systems impact on gender inequalities in pain prevalence.

## Conclusions

This study provides the first comprehensive overview of gender pain inequalities across Europe. The most common type of pain experienced by both men and women was back/neck pain. At the pan-European level, significant gender pain inequalities exist whereby women experience more pain than men. While this was found at a pan-European level, the extent of the gender pain inequality varied by country, as different degrees of inequality in pain were reported by different countries. This gender pain divide is a public health concern and it should be considered in any future pain prevention and management strategies going forward.

## Supplemental Material

sj-pdf-1-sjp-10.1177_1403494820987466 – Supplemental material for The Gender Pain Gap: gender inequalities in pain across 19 European countries Click here for additional data file. Supplemental material, sj-pdf-1-sjp-10.1177_1403494820987466 for The Gender Pain Gap: gender inequalities in pain across 19 European countries by Kweku Bimpong, Katie Thomson, Courtney L. Mcnamara, Mirza Balaj, Nasima Akhter, Clare Bambra and Adam Todd in Scandinavian Journal of Public Health

sj-pdf-2-sjp-10.1177_1403494820987466 – Supplemental material for The Gender Pain Gap: gender inequalities in pain across 19 European countries Click here for additional data file. Supplemental material, sj-pdf-2-sjp-10.1177_1403494820987466 for The Gender Pain Gap: gender inequalities in pain across 19 European countries by Kweku Bimpong, Katie Thomson, Courtney L. Mcnamara, Mirza Balaj, Nasima Akhter, Clare Bambra and Adam Todd in Scandinavian Journal of Public Health

sj-pdf-3-sjp-10.1177_1403494820987466 – Supplemental material for The Gender Pain Gap: gender inequalities in pain across 19 European countries Click here for additional data file. Supplemental material, sj-pdf-3-sjp-10.1177_1403494820987466 for The Gender Pain Gap: gender inequalities in pain across 19 European countries by Kweku Bimpong, Katie Thomson, Courtney L. Mcnamara, Mirza Balaj, Nasima Akhter, Clare Bambra and Adam Todd in Scandinavian Journal of Public Health
