# Supplementary material for: The Gender Pain Gap: gender inequalities in pain across 19 European countries
Source: Scand J Public Health. 2021 Feb 10;50(2):287–94. doi: 10.1177/1403494820987466 (PMC8873965; doi:10.1177/1403494820987466)
Supplement: sj-pdf-1-sjp-10.1177_1403494820987466 – Supplemental material for The Gender Pain Gap: gender inequalities in pain across 19 European countries [file sj-pdf-1-sjp-10.1177_1403494820987466.pdf]

Supplementary Table 1: A sensitivity analysis showing prevalence of pain by gender at the pan-European level.

Blue shows pain prevalence for participants in the 25-74 years age range; while red shows pain prevalence for participants in the 15+ years age range.

**Pain Variable**		**Back/Neck (%)**	**Hand/Arm (%)**	**Foot/leg (%)**	**Any pain (%)**
**Europe (pooled)**	Men	40.8	22.8	24.3	55.5
		38.3	21.8	24.5	54.6
	Difference	**2.5**	**1.0**	**-0.2**	**0.9**
	Women	47.3	27.4	26.6	62.3
		**46.2**	**27**	**27.8**	**62.2**
	Difference	**1.1**	**0.4**	**-1.2**	**0.1**
