# Supplementary material for: The Gender Pain Gap: gender inequalities in pain across 19 European countries
Source: Scand J Public Health. 2021 Feb 10;50(2):287–94. doi: 10.1177/1403494820987466 (PMC8873965; doi:10.1177/1403494820987466)
Supplement: sj-pdf-2-sjp-10.1177_1403494820987466 – Supplemental material for The Gender Pain Gap: gender inequalities in pain across 19 European countries [file sj-pdf-2-sjp-10.1177_1403494820987466.pdf]

Supplementary Table 2: an overview of missing data

Cases deleted for missing data of: country, pain variables, gender, age

	Initial sample	40185
Restriction	Drop Israel	2562
	Drop Estonia	2051
	Drop Age <25	4111
	Drop Age >=74	3,387
Total restriction	Selected variables	12,111
Missing	pain variables	296
	gender	15
	Age	72
Total missing cases		383
Total case including missing cases		27,935
% missing cases		1.4%
	Final sample	27552
