# Supplementary material for: The Gender Pain Gap: gender inequalities in pain across 19 European countries
Source: Scand J Public Health. 2021 Feb 10;50(2):287–94. doi: 10.1177/1403494820987466 (PMC8873965; doi:10.1177/1403494820987466)
Supplement: sj-pdf-3-sjp-10.1177_1403494820987466 – Supplemental material for The Gender Pain Gap: gender inequalities in pain across 19 European countries [file sj-pdf-3-sjp-10.1177_1403494820987466.pdf]

Supplementary Table 3. Gender pain inequalities for total pain in 19 European Countries calculated using different models

	Country	Linear Probability model	Binary Logistic Regression model	
		Coefficient (95% confidence intervals)	ARD (95% confidence intervals)	ARR (95% confidence intervals)
**Europe (pooled)**		0.062 (0.017, 0.104)	5.5% (4.1%, 6.9%)	1.10 (1.08, 1.13)
**North**	Denmark	0.037 (-0.020, 0.090)	3.0% (-3.1%, 9.2%)	1.05 (0.95, 1.15)
	Finland	0.105 (0.050, 0.139)	9.9% (5.0%, 14.9%)	1.15 (1.07, 1.23)
	Norway	0.117 (0.056, 0.169)	10.7% (4.8%, 16.7%)	1.18 (1.08, 1.30)
	Sweden	0.059 (0.007, 0.112)	6.2% (0.8%, 11.7%)	1.10 (1.01, 1.20)
**West**	Austria	0.018 (-0.034, 0.070)	0.9% (-4.3%, 6.2%)	1.02 (0.90, 1.15)
	Belgium	0.055 (0.00, 0.102)	5.0% (-0.3%, 10.2%)	1.07 (1.00, 1.16)
	Switzerland	0.059 (0.007, 0.112)	3.7% (-2.0%, 9.6%)	1.07 (0.97, 1.18)
	Germany	0.080 (0.035, 0.112)	7.6% (3.3%, 11.9%)	1.12 (1.05, 1.19)
	France	0.106 (0.050, 0.146)	9.9% (3.8%, 16.0%)	1.16 (1.06, 1.27)
	Ireland	0.006 (-0.038, 0.049)	6.1% (0.3%, 11.9%)	1.18 (1.00, 1.38)
	Netherlands	0.065 (0.013, 0.114)	6.6% (0.6%, 12.6%)	1.12 (1.01, 1.24)
	United Kingdom	0.004 (-0.042, 0.051)	0.2% (-5.1%, 5.5%)	1.00 (0.91, 1.10)
**Central/ Eastern**	Poland	0.069 (0.013, 0.114)	5.7% (-0.1%, 11.5%)	1.12 (1.00, 1.27)
	Slovenia	0.127 (0.061, 0.190)	12.4% (5.3%, 19.4%)	1.25 (1.09, 1.42)
	Lithuania	0.067 (0.017, 0.113)	6.1% (0.3%, 11.9%)	1.18 (1.00, 1.38)
	Czech Republic	0.096 (0.045, 0.139)	8.8% (3.7%, 13.8%)	1.29 (1.11, 1.49)
	Hungary	0.049 (-0.007, 0.094)	2.5% (-3.0%, 7.9%)	1.10 (0.89, 1.34)
**South**	Spain	0.107 (0.054, 0.157)	11.2% (5.8%, 16.5%)	1.22 (1.10, 1.34)
	Portugal	0.045 (-0.0019, 0.104)	4.1% (-4.0%, 12.2%)	1.06 (0.94, 1.20)

Table 3: Linear Probability model coefficients, and Binary logistic regression generated age-adjusted rate differences (ARD) and age-adjusted rate ratios (ARR) for gender inequalities in back/neck pain, hand/arm pain, and foot/leg pain in 19 European Countries. Pain in men was the reference group. Linear Probability Model Coefficient and ARD estimated whether absolute difference in prevalence between male and female is statistically different to 0, while ARR assessed if the rate ratio is significantly different to 1 (where 1 is equal risk). Pain inequality was defined where the ARD and ARR were both statistically significant.
